# Triaqua­chlorido[3-dimethyl­amino-1-(2-pyrid­yl)prop-2-en-1-one-κ*N* ^1^]manganese(II) chloride

**DOI:** 10.1107/S1600536809024192

**Published:** 2009-07-01

**Authors:** Zhao-Lian Chu

**Affiliations:** a School of Chemistry and Chemical Engineering, Anhui University of Technology, Maanshan 243002, People’s Republic of China

## Abstract

In the title compound, [MnCl(C_10_H_12_N_2_O)(H_2_O)_3_]Cl, the Mn^II^ ion has a distorted octa­hedral coordination environment formed by one N and one O atom from the chelating 3-dimethyl­amino-1-(2-pyrid­yl)prop-2-en-1-one ligand, one chloride anion and three coordinated water mol­ecules. Inter­molecular O—H⋯O and O—H⋯Cl hydrogen bonds link the cations and anions into layers parallel to the *ac* plane.

## Related literature

For the crystal structure of a related Cd(II) complex, see: Dong *et al.* (2009 ▶). For details of the synthesis, see: Sun *et al.* (2008 ▶).
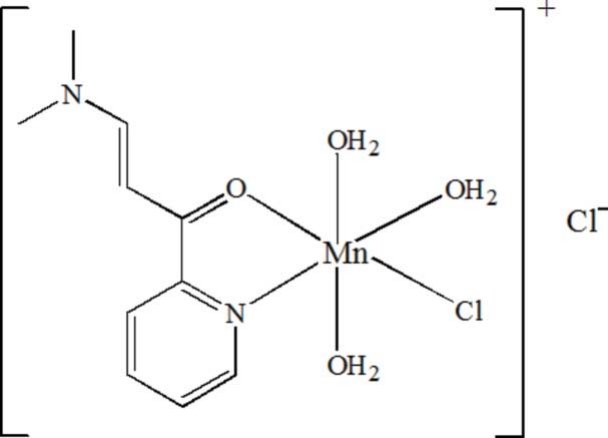


## Experimental

### 

#### Crystal data

[MnCl(C_10_H_12_N_2_O)(H_2_O)_3_]Cl
*M* *_r_* = 356.10
Triclinic, 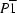

*a* = 8.7039 (17) Å
*b* = 9.3247 (18) Å
*c* = 10.1407 (19) Å
α = 98.029 (4)°
β = 98.036 (4)°
γ = 107.357 (3)°
*V* = 763.4 (3) Å^3^
*Z* = 2
Mo *K*α radiation
μ = 1.22 mm^−1^
*T* = 291 K
0.30 × 0.20 × 0.20 mm

#### Data collection

Bruker SMART CCD area-detector diffractometer
Absorption correction: multi-scan (*SADABS*; Bruker, 2000 ▶) *T*_min_ = 0.710, *T*_max_ = 0.792
3838 measured reflections
2647 independent reflections
1898 reflections with *I* > 2σ(*I*)
*R*_int_ = 0.026

#### Refinement

*R*[*F* ^2^ > 2σ(*F* ^2^)] = 0.048
*wR*(*F* ^2^) = 0.096
*S* = 0.90
2647 reflections
174 parameters
H-atom parameters constrained
Δρ_max_ = 0.37 e Å^−3^
Δρ_min_ = −0.36 e Å^−3^

### 

Data collection: *SMART* (Bruker, 2000 ▶); cell refinement: *SAINT* (Bruker, 2000 ▶); data reduction: *SAINT*; program(s) used to solve structure: *SHELXS97* (Sheldrick, 2008 ▶); program(s) used to refine structure: *SHELXL97* (Sheldrick, 2008 ▶); molecular graphics: *SHELXTL* (Sheldrick, 2008 ▶); software used to prepare material for publication: *publCIF* (Westrip, 2009 ▶).

## Supplementary Material

Crystal structure: contains datablocks I, global. DOI: 10.1107/S1600536809024192/cv2579sup1.cif

Structure factors: contains datablocks I. DOI: 10.1107/S1600536809024192/cv2579Isup2.hkl

Additional supplementary materials:  crystallographic information; 3D view; checkCIF report

## Figures and Tables

**Table 1:** Hydrogen-bond geometry (Å, °)

*D*—H⋯*A*	*D*—H	H⋯*A*	*D*⋯*A*	*D*—H⋯*A*
O2—H2*B*⋯Cl1^i^	0.85	2.58	3.142 (3)	125
O2—H2*C*⋯Cl2	0.85	2.64	3.188 (3)	124
O3—H3*B*⋯Cl2^ii^	0.85	2.46	3.228 (3)	150
O3—H3*C*⋯Cl2^iii^	0.85	2.48	3.090 (3)	129
O4—H4*B*⋯O1^ii^	0.85	2.27	2.659 (3)	108
O4—H4*C*⋯Cl2	0.85	2.41	3.063 (3)	134

Symmetry codes: (i) 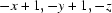; (ii) 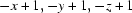; (iii) 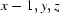.

**Fig. 1.**
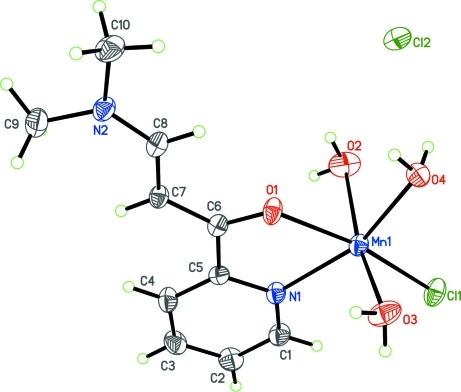
View of (I) showing 30% probability displacement ellipsoids and the atomic numbering.

## supplementary crystallographic information

### Comment

We have taken many efforts on synthesizing new ligands with pyridyl group and reported a monomeric Cd (II) complex using 3-dimethylamino-1-(4-pyridyl-)prop-2-en-1-one as ligand (Dong *et al.*, 2009). Here we obtain an analogous ligand, 3-dimethylamino-1-(2-pyridyl-)prop-2-en-1-one by similar method, and report a new Mn (II) complex, *viz.* the title compound, [Mn(C_10_H_12_N_2_O)(H_2_O)_3_Cl]^+^.Cl^-^ (I).

In (I) (Fig. 1), the Mn^II^ center shows an octahedral coordination geometry formed by NO_4_Cl. Cholride anions are involved in formation of O—H···Cl hydrogen bonds (Table 1), which link cations and anions into layers parallel to *ac* plane along with the intermolecular O—H···O hydrogen bonds (Table 1).

### Experimental

Ligand was prepared following the procedure reported in the literature (Sun *et al.*, 2008). A solution of the ligand (0.1 mmol) and MnCl_2_ (0.1 mmol) in 40 ml of methanol was refluxed for 2 h, and then cooled to room temperature and filtered. Single crystals suittable for X-ray analysis were grown from the methanol solution by slow evaporation at room temperature in air. Anal. Calcd. for C_10_H_18_MnN_2_O_4_Cl_2_: C, 33.72; H, 5.09; N, 7.87. Found: C, 33.68; H, 5.13; N, 7.83.

### Refinement

All hydrogen atoms were geometrically positioned (C—H 0.93–0.97 Å, O–H 0.85 Å) and refined as riding, with *U*_iso_(H)=1.2–1.5 *U*_eq_ of the parent atom.

### Crystal data

**Table:**

[MnCl(C_10_H_12_N_2_O)(H_2_O)_3_]Cl	*Z* = 2
*M**_r_* = 356.10	*F*(000) = 366
Triclinic, *P*1	*D*_x_ = 1.549 Mg m^−^^3^
Hall symbol: -P 1	Mo *K*α radiation, λ = 0.71073 Å
*a* = 8.7039 (17) Å	Cell parameters from 956 reflections
*b* = 9.3247 (18) Å	θ = 2.3–27.9°
*c* = 10.1407 (19) Å	µ = 1.22 mm^−^^1^
α = 98.029 (4)°	*T* = 291 K
β = 98.036 (4)°	Block, colourless
γ = 107.357 (3)°	0.30 × 0.20 × 0.20 mm
*V* = 763.4 (3) Å^3^	

### Data collection

**Table:**

Bruker SMART CCD area-detector diffractometer	2647 independent reflections
Radiation source: fine-focus sealed tube	1898 reflections with *I* > 2σ(*I*)
graphite	*R*_int_ = 0.026
φ and ω scans	θ_max_ = 25.0°, θ_min_ = 2.1°
Absorption correction: multi-scan (*SADABS*; Bruker, 2000)	*h* = −8→10
*T*_min_ = 0.710, *T*_max_ = 0.792	*k* = −10→11
3838 measured reflections	*l* = −12→7

### Refinement

**Table:**

Refinement on *F*^2^	Primary atom site location: structure-invariant direct methods
Least-squares matrix: full	Secondary atom site location: difference Fourier map
*R*[*F*^2^ > 2σ(*F*^2^)] = 0.048	Hydrogen site location: inferred from neighbouring sites
*wR*(*F*^2^) = 0.096	H-atom parameters constrained
*S* = 0.90	*w* = 1/[σ^2^(*F*_o_^2^) + (0.0358*P*)^2^] where *P* = (*F*_o_^2^ + 2*F*_c_^2^)/3
2647 reflections	(Δ/σ)_max_ < 0.001
174 parameters	Δρ_max_ = 0.37 e Å^−^^3^
0 restraints	Δρ_min_ = −0.36 e Å^−^^3^

### Special details

**Table:**

Experimental. The structure was solved by direct methods (Bruker, 2000) and successive difference Fourier syntheses.
Geometry. All e.s.d.'s (except the e.s.d. in the dihedral angle between two l.s. planes) are estimated using the full covariance matrix. The cell e.s.d.'s are taken into account individually in the estimation of e.s.d.'s in distances, angles and torsion angles; correlations between e.s.d.'s in cell parameters are only used when they are defined by crystal symmetry. An approximate (isotropic) treatment of cell e.s.d.'s is used for estimating e.s.d.'s involving l.s. planes.
Refinement. Refinement of *F*^2^ against ALL reflections. The weighted *R*-factor *wR* and goodness of fit *S* are based on *F*^2^, conventional *R*-factors *R* are based on *F*, with *F* set to zero for negative *F*^2^. The threshold expression of *F*^2^ > σ(*F*^2^) is used only for calculating *R*-factors(gt) *etc*. and is not relevant to the choice of reflections for refinement. *R*-factors based on *F*^2^ are statistically about twice as large as those based on *F*, and *R*- factors based on ALL data will be even larger.

### Fractional atomic coordinates and isotropic or equivalent isotropic displacement parameters (Å^2^)

**Table:**

	*x*	*y*	*z*	*U*_iso_*/*U*_eq_	
Mn1	0.38327 (7)	0.51169 (6)	0.24526 (6)	0.0323 (2)	
Cl1	0.18927 (14)	0.33243 (12)	0.05540 (10)	0.0480 (3)	
Cl2	0.81678 (13)	0.36167 (13)	0.32531 (11)	0.0498 (3)	
N1	0.3684 (4)	0.7276 (3)	0.1810 (3)	0.0331 (8)	
N2	0.9228 (4)	1.0462 (4)	0.6561 (3)	0.0379 (9)	
O1	0.5460 (3)	0.7046 (3)	0.4013 (2)	0.0390 (7)	
O2	0.6042 (3)	0.5194 (3)	0.1505 (3)	0.0602 (9)	
H2B	0.6189	0.5984	0.1148	0.072*	
H2C	0.6781	0.5474	0.2219	0.072*	
O3	0.1955 (3)	0.4766 (3)	0.3751 (3)	0.0536 (8)	
H3B	0.2274	0.5420	0.4489	0.064*	
H3C	0.1121	0.4840	0.3255	0.064*	
O4	0.4698 (3)	0.3588 (3)	0.3523 (2)	0.0391 (7)	
H4B	0.5335	0.4114	0.4257	0.047*	
H4C	0.5278	0.3238	0.3042	0.047*	
C1	0.2670 (5)	0.7350 (5)	0.0724 (4)	0.0408 (11)	
H1	0.1950	0.6441	0.0189	0.049*	
C2	0.2641 (5)	0.8704 (5)	0.0362 (4)	0.0471 (12)	
H2A	0.1909	0.8712	−0.0396	0.056*	
C3	0.3706 (5)	1.0043 (5)	0.1136 (4)	0.0481 (12)	
H3A	0.3712	1.0979	0.0914	0.058*	
C4	0.4769 (5)	0.9982 (4)	0.2251 (4)	0.0415 (11)	
H4A	0.5509	1.0880	0.2787	0.050*	
C5	0.4732 (5)	0.8596 (4)	0.2565 (4)	0.0305 (9)	
C6	0.5787 (5)	0.8396 (4)	0.3796 (3)	0.0303 (9)	
C7	0.7021 (5)	0.9654 (4)	0.4608 (4)	0.0325 (10)	
H7	0.7174	1.0630	0.4420	0.039*	
C8	0.8019 (5)	0.9433 (4)	0.5698 (4)	0.0363 (10)	
H8	0.7799	0.8430	0.5831	0.044*	
C9	0.9737 (6)	1.2080 (4)	0.6504 (4)	0.0541 (13)	
H9A	0.9783	1.2201	0.5585	0.081*	
H9B	1.0802	1.2584	0.7066	0.081*	
H9C	0.8963	1.2523	0.6826	0.081*	
C10	1.0158 (5)	1.0020 (5)	0.7672 (4)	0.0507 (12)	
H10A	0.9657	0.8953	0.7675	0.076*	
H10B	1.0157	1.0618	0.8523	0.076*	
H10C	1.1267	1.0200	0.7543	0.076*	

### Atomic displacement parameters (Å^2^)

**Table:**

	*U*^11^	*U*^22^	*U*^33^	*U*^12^	*U*^13^	*U*^23^
Mn1	0.0342 (4)	0.0306 (4)	0.0272 (4)	0.0057 (3)	0.0003 (3)	0.0059 (3)
Cl1	0.0504 (7)	0.0436 (7)	0.0337 (6)	−0.0023 (5)	−0.0019 (5)	0.0035 (5)
Cl2	0.0352 (6)	0.0591 (7)	0.0507 (7)	0.0126 (6)	0.0047 (5)	0.0055 (6)
N1	0.037 (2)	0.0306 (18)	0.0264 (18)	0.0074 (16)	−0.0017 (15)	0.0038 (15)
N2	0.036 (2)	0.037 (2)	0.033 (2)	0.0064 (17)	−0.0017 (16)	0.0013 (16)
O1	0.0468 (19)	0.0289 (16)	0.0298 (16)	0.0009 (14)	−0.0060 (13)	0.0064 (12)
O2	0.052 (2)	0.088 (2)	0.0515 (19)	0.0239 (19)	0.0188 (16)	0.0382 (18)
O3	0.0363 (19)	0.077 (2)	0.0376 (18)	0.0129 (17)	0.0030 (14)	−0.0036 (15)
O4	0.0437 (18)	0.0388 (16)	0.0350 (16)	0.0152 (14)	0.0051 (13)	0.0061 (13)
C1	0.041 (3)	0.042 (3)	0.033 (2)	0.009 (2)	−0.004 (2)	0.006 (2)
C2	0.052 (3)	0.055 (3)	0.035 (3)	0.022 (3)	−0.003 (2)	0.015 (2)
C3	0.057 (3)	0.042 (3)	0.051 (3)	0.021 (2)	0.008 (2)	0.019 (2)
C4	0.050 (3)	0.030 (2)	0.039 (3)	0.007 (2)	0.003 (2)	0.008 (2)
C5	0.029 (2)	0.035 (2)	0.027 (2)	0.0113 (19)	0.0056 (17)	0.0044 (18)
C6	0.033 (2)	0.033 (2)	0.023 (2)	0.0069 (19)	0.0096 (17)	0.0018 (18)
C7	0.036 (2)	0.029 (2)	0.027 (2)	0.0060 (19)	0.0019 (18)	0.0030 (17)
C8	0.035 (3)	0.035 (2)	0.033 (2)	0.003 (2)	0.0072 (19)	0.0021 (19)
C9	0.054 (3)	0.038 (3)	0.057 (3)	0.008 (2)	−0.003 (2)	−0.001 (2)
C10	0.046 (3)	0.059 (3)	0.039 (3)	0.013 (2)	−0.007 (2)	0.007 (2)

### Geometric parameters (Å, °)

**Table:**

Mn1—O4	2.150 (2)	C1—H1	0.9300
Mn1—O1	2.192 (2)	C2—C3	1.366 (5)
Mn1—O3	2.217 (3)	C2—H2A	0.9300
Mn1—N1	2.234 (3)	C3—C4	1.377 (5)
Mn1—O2	2.253 (3)	C3—H3A	0.9300
Mn1—Cl1	2.4208 (11)	C4—C5	1.366 (5)
N1—C1	1.335 (4)	C4—H4A	0.9300
N1—C5	1.344 (4)	C5—C6	1.511 (5)
N2—C8	1.303 (4)	C6—C7	1.392 (5)
N2—C9	1.452 (5)	C7—C8	1.387 (5)
N2—C10	1.473 (5)	C7—H7	0.9300
O1—C6	1.263 (4)	C8—H8	0.9300
O2—H2B	0.8500	C9—H9A	0.9600
O2—H2C	0.8501	C9—H9B	0.9600
O3—H3B	0.8500	C9—H9C	0.9600
O3—H3C	0.8498	C10—H10A	0.9600
O4—H4B	0.8500	C10—H10B	0.9600
O4—H4C	0.8500	C10—H10C	0.9600
C1—C2	1.369 (5)		
			
O4—Mn1—O1	89.04 (9)	C3—C2—C1	118.8 (4)
O4—Mn1—O3	84.40 (11)	C3—C2—H2A	120.6
O1—Mn1—O3	89.54 (10)	C1—C2—H2A	120.6
O4—Mn1—N1	160.52 (10)	C2—C3—C4	118.8 (4)
O1—Mn1—N1	72.14 (10)	C2—C3—H3A	120.6
O3—Mn1—N1	100.02 (12)	C4—C3—H3A	120.6
O4—Mn1—O2	81.56 (10)	C5—C4—C3	119.7 (4)
O1—Mn1—O2	86.99 (11)	C5—C4—H4A	120.1
O3—Mn1—O2	165.59 (10)	C3—C4—H4A	120.1
N1—Mn1—O2	92.22 (11)	N1—C5—C4	121.8 (3)
O4—Mn1—Cl1	100.99 (7)	N1—C5—C6	114.0 (3)
O1—Mn1—Cl1	169.97 (8)	C4—C5—C6	124.2 (3)
O3—Mn1—Cl1	91.26 (8)	O1—C6—C7	124.3 (3)
N1—Mn1—Cl1	97.88 (8)	O1—C6—C5	115.6 (3)
O2—Mn1—Cl1	94.59 (8)	C7—C6—C5	120.1 (3)
C1—N1—C5	118.0 (3)	C8—C7—C6	119.1 (4)
C1—N1—Mn1	125.2 (3)	C8—C7—H7	120.4
C5—N1—Mn1	116.8 (2)	C6—C7—H7	120.4
C8—N2—C9	123.4 (3)	N2—C8—C7	127.8 (4)
C8—N2—C10	120.5 (3)	N2—C8—H8	116.1
C9—N2—C10	116.1 (3)	C7—C8—H8	116.1
C6—O1—Mn1	120.2 (2)	N2—C9—H9A	109.5
Mn1—O2—H2B	103.2	N2—C9—H9B	109.5
Mn1—O2—H2C	99.5	H9A—C9—H9B	109.5
H2B—O2—H2C	104.5	N2—C9—H9C	109.5
Mn1—O3—H3B	111.7	H9A—C9—H9C	109.5
Mn1—O3—H3C	103.7	H9B—C9—H9C	109.5
H3B—O3—H3C	112.7	N2—C10—H10A	109.5
Mn1—O4—H4B	107.9	N2—C10—H10B	109.5
Mn1—O4—H4C	106.9	H10A—C10—H10B	109.5
H4B—O4—H4C	106.9	N2—C10—H10C	109.5
N1—C1—C2	123.0 (4)	H10A—C10—H10C	109.5
N1—C1—H1	118.5	H10B—C10—H10C	109.5
C2—C1—H1	118.5		

### Hydrogen-bond geometry (Å, °)

**Table:**

*D*—H···*A*	*D*—H	H···*A*	*D*···*A*	*D*—H···*A*
O2—H2B···Cl1^i^	0.85	2.58	3.142 (3)	125
O2—H2C···Cl2	0.85	2.64	3.188 (3)	124
O3—H3B···Cl2^ii^	0.85	2.46	3.228 (3)	150
O3—H3C···Cl2^iii^	0.85	2.48	3.090 (3)	129
O4—H4B···O1^ii^	0.85	2.27	2.659 (3)	108
O4—H4C···Cl2	0.85	2.41	3.063 (3)	134

Symmetry codes: (i) −*x*+1, −*y*+1, −*z*; (ii) −*x*+1, −*y*+1, −*z*+1; (iii) *x*−1, *y*, *z*.

## References

[bb1] Bruker (2000). *SADABS*, *SMART* and *SAINT* Bruker AXS Inc., Madison, Wisconsin, USA.

[bb2] Dong, H.-Z., Chu, Z.-L. & Hu, N.-L. (2009). *Acta Cryst.* E**65**, m358. 10.1107/S1600536809007028 PMC2968920 21582316

[bb3] Sheldrick, G. M. (2008). *Acta Cryst.* A**64**, 112–122. 10.1107/S0108767307043930 18156677

[bb4] Sun, Y.-Y., Dong, H.-Z. & Cheng, L. (2008). *Acta Cryst.* E**64**, o901. 10.1107/S1600536808010854 PMC2961214 21202384

[bb5] Westrip, S. P. (2009). *publCIF* In preparation.

